# Case of catastrophic antiphospholipid syndrome presenting as neuroretinitis and vaso-occlusive retinopathy

**DOI:** 10.1186/s12886-020-01755-9

**Published:** 2020-12-09

**Authors:** Young In Yun, Ji Hyun Kim, Seon Hee Lim, Yo Han Ahn, Hee Gyung Kang, Il-Soo Ha, Baek-Lok Oh

**Affiliations:** 1 Department of Ophthalmology, Seoul National University Hospital, 101 Daehak-ro, Jongno-gu, Seoul, 03080 South Korea; 2 Department of Pediatrics, Seoul National University Bundang Hospital, Seongnam, South Korea; 3 Department of Pediatrics, Seoul National University Children’s Hospital, Seoul, South Korea

**Keywords:** Catastrophic antiphospholipid syndrome, Antiphospholipid syndrome, Neuroretinitis, Vaso-occlusive retinopathy, Thrombotic microangiopathy

## Abstract

**Background:**

Ocular involvement in catastrophic antiphospholipid syndrome (CAPS), a rare, life-threatening form of antiphospholipid syndrome (APS) that results in multiorgan failure and a high mortality rate, has rarely been reported.

**Case presentation:**

A 15-year-old girl presented with sudden vision blurring in both eyes. She had marked optic disc swelling and macular exudates in the right eye and intra-arterial white plaques, a few retinal blot hemorrhages, and a white ischemic retina in the left eye. Systemic examination revealed she had acute kidney injury with thrombotic microangiopathy (TMA), multiple cerebral infarcts, valvular dysfunction, and a high titer of triple aPL. Thus, she was diagnosed with CAPS involving the brain, eyes, heart, and kidneys. Plasma exchange and the administration of glucocorticoids, immunoglobulin, warfarin, and rituximab brought a sustained recovery of the TMA, visual symptoms, and echocardiographic findings.

**Conclusions:**

Ocular involvement of both vaso-occlusive retinopathy, an APS-related thrombotic microangiopathy, and neuroretinitis, a non-thrombotic microangiopathy, can occur as an initial presentation of CAPS.

## Background

Antiphospholipid syndrome (APS) is a systemic autoimmune disease characterized by arterial and venous thrombosis that is induced by antiphospholipid antibodies (aPL): lupus anticoagulant (LA), anticardiolipin, and anti-beta2-glycoprotein I (anti-β2-GPI). Catastrophic APS (CAPS) is a rare, life-threatening form of APS that results in multiorgan failure and a high mortality rate.

The most frequently involved sites in CAPS are the kidneys (73%), lungs (60%), brain (56%), and heart (50%) [1, 2]. Ocular involvement in CAPS has rarely been reported [3–5]. We report the first case of CAPS in an adolescent girl with concurrent vaso-occlusive retinopathy and neuroretinitis, which may reflect microangiopathies from thrombotic and non-thrombotic pathophysiology of APS, respectively.

## Case presentation

A 15-year-old girl presented to Seoul National University Hospital on December 17, 2018 with sudden vision blurring in both eyes that started 8 days prior. She previously had episodes of transient right arm weakness and dysarthria 22 and 6 months  before, respectively, but no medical attention was sought. She was not on any medication, and ophthalmic history was negative. At presentation, she had dysuria, frequency, urgency and gross hematuria with mild fever. Her blood pressure was 171/135 mmHg. She was otherwise healthy and no family or trauma history was noted.

**Fig. 1:**
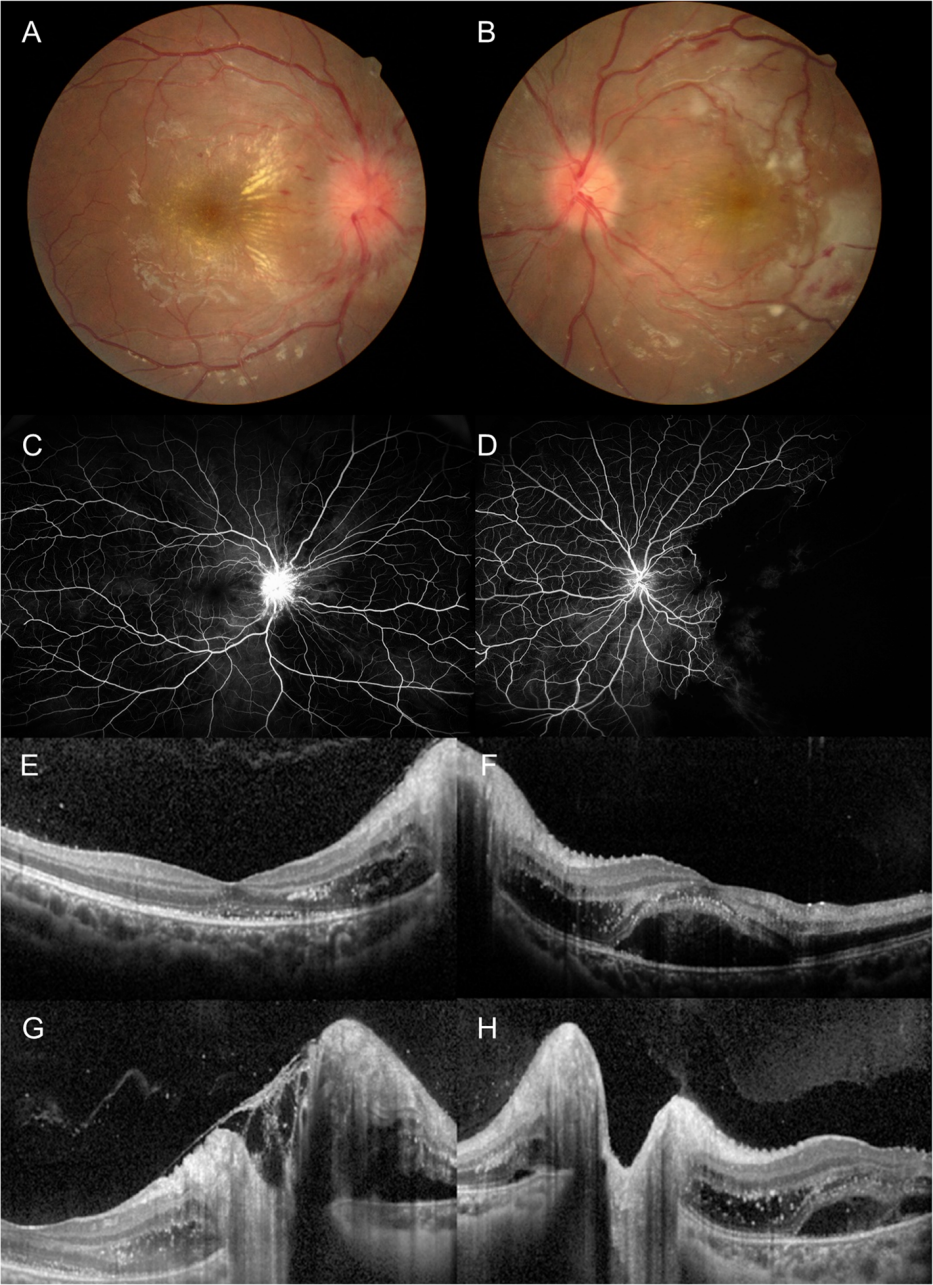
Ocular manifestations of a pediatric catastrophic anti-phospholipid syndrome. **a** Severe optic disc swelling, multiple blot hemorrhage, and macular exudates are seen in the right eye. **b** Optic disc swelling, macular exudates, multiple hemorrhages, intraarterial white plaques and white ischemic retina are observed in the left eye. **c**, **d**, Fluorescein angiographies revealed disc and vascular leakage with decreased choroidal perfusion are found in both eyes, and large non-perfusion area at temporal retina in the left eye. **e**, **f**, **g**, **h** Optical coherence tomography showed fluid and multiple hard exudates in subretinal space and Henle’s layer at macula and peripapillary area. Bilateral optic disc edema is observed

Her corrected visual acuities were 20/50 in the right eye and 20/35 in the left eye. Ophthalmic examination revealed marked optic disc swelling and macular exudates in the right eye (Fig. 1a). In the left eye, in addition to mild disc swelling and macular exudates, intra-arterial white plaques, a few retinal blot hemorrhages, and a white ischemic retina were observed (Fig. 1b). Fluorescein angiographies revealed disc and vascular leakage with decreased choroidal perfusion in both eyes, and retinal arterial occlusions with large non-perfusion area at temporal retina was observed in the left eye (Fig. 1c and d). Optical coherence tomography (OCT) images confirmed bilateral disc edema and showed fluid and multiple hard exudates in subretinal space and Henle’s layer at macula and peripapillary area in both eyes (Fig. 1e-h).

Laboratory tests revealed microangiopathic hemolytic anemia with thrombocytopenia; hemoglobin 8.7 g/dL, platelet 60,000/uL, haptoglobin < 7 mg/dL, with positive schistocytes (2.1/HPF). C-reactive protein was elevated (5.93 mg/dL) and complements were marginally decreased (C3/C4 80/6 mg/dL [normal range: 83-193 mg/dL/15-57 mg/dL]), but antinuclear antibody, anti-dsDNA, anti-SSA and anti-SSB were negative. She showed positive LA with high titers of anti-β2-GPI (IgM/IgG 225.6/140.7 IU/mL [normal range: negative (< 20 IU/mL) in both]) and anticardiolipin antibodies (IgM/IgG 90.5/159.6 IU/mL). Renal ultrasonography showed acute pyelonephritis and acute kidney injury, and kindey biopsy findings were compatible with thrombotic microangiopathy (TMA). In addition, valvular dysfunction was noted in echocardiography. Brain MRI showed multiple infarction at right periventricular and subcortical area and left frontal lobe. Serologic studies for toxoplasma, hepatitis B, human immunodeficiency virus, and syphilis revealed negative. Therefore, she was diagnosed with primary CAPS, involving the brain, heart, kidneys, and the eyes. Although her blood pressure was relatively high, the optic disc swellings were not regarded as a manifestation of stage 4 hypertensive retinopathy due to the inter-eye asymmetry of disc swellings and the absence of characteristic findings of a stage 4 hypertensive retinopathy, including sclerotic vessel changes, flame-shaped hemorrhages and cotton-wool spots centered on the optic disc.

**Fig. 2:**
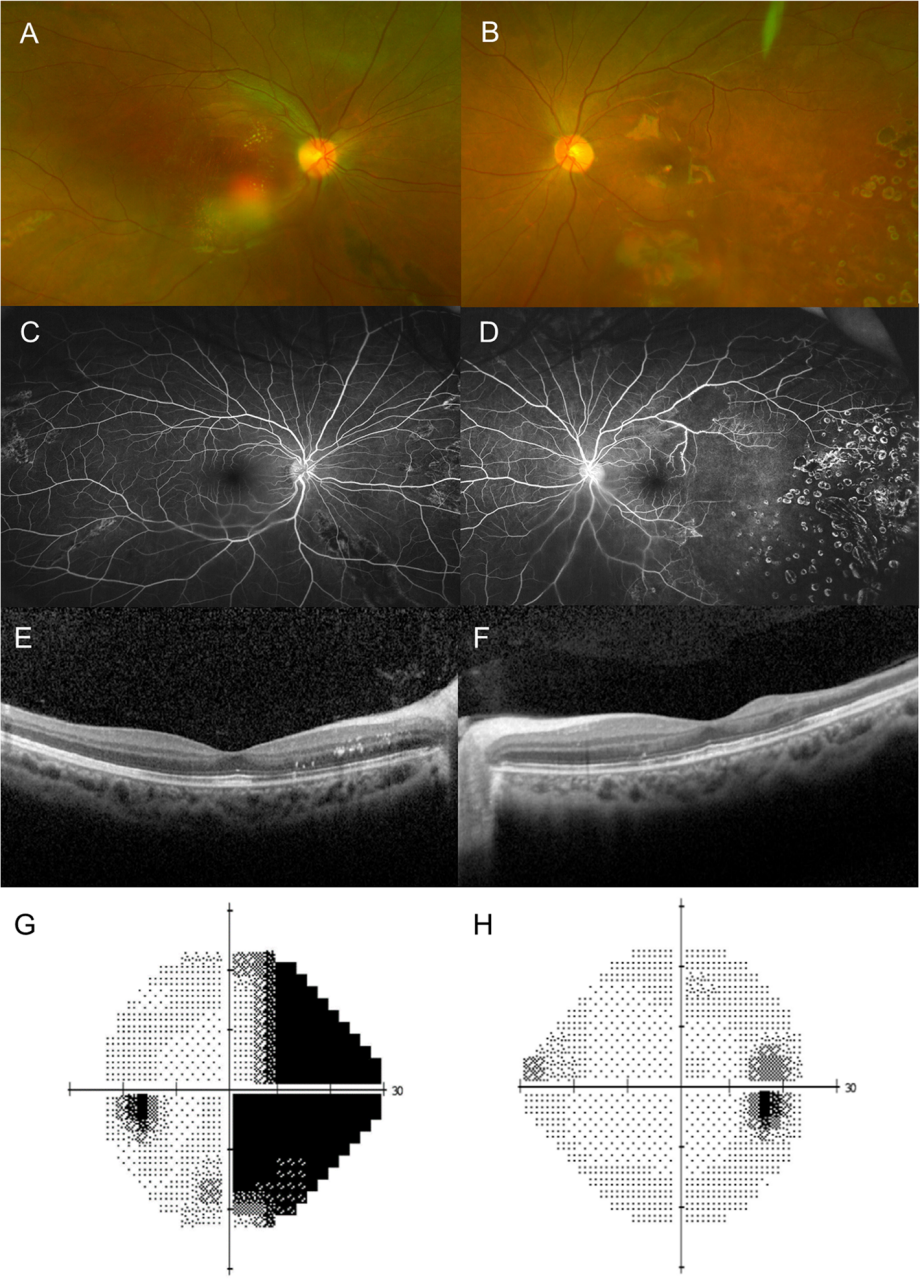
Ocular findings of pediatric catastrophic anti-phospholipid syndrome at 6 months after presentation. **a** The fundus showed no optic disc edema, but a few remnant exudates are seen in the right eye. **b** Occluded retinal arteries and laser marks at temporal retinal area are observed in the left eye. **c**, **d** Fluorescein angiographies revealed no disc leakage in the both eyes. Although there is non-perfused retinal area, no active retinal neovascularization is observed. **e**, **f** Optical coherence tomography of macula showed flat macula with a few remnant exudates are detected in the both eyes. Temporal retinal thinning is observed in the left eye. **g** Nasal field defect is shown in the left eye. **h** No definite visual field defect is detected in the right eye

Plasma exchange and the administration of glucocorticoids, intravenous immunoglobulin (IVIG), warfarin, and rituximab brought a sustained recovery of the TMA, visual symptoms, and echocardiographic findings. Retinal scatter laser treatments were performed in her left eye to prevent neovascularization of the avascular retina. Six months after presentation, her corrected visual acuities were 20/20 in both eyes, and the fundus showed no disc edema with few remnant exudates (Fig. 2a, b). OCT scans revealed superior and temporal retinal thinning in the left eye (Fig. 2f). Visual field test showed a nasal field defect in the left eye, which was consistent with the non-perfused area in the temporal retina (Fig. 2g).

## Discussion and conclusions

CAPS is associated with a high mortality rate; therefore, early detection and aggressive therapy are very important [6, 7]. Currently, the expert consensus recommends the use of the so-called ‘triple therapy’ for CAPS: glucocorticoids, plasma exchange or IVIG, and anticoagulation therapy regardless of the severity of thrombocytopenia [1, 8–10]. The exact pathogenesis of APS/CAPS has not been fully elucidated. However, both a thrombotic mechanism, which is caused by the aPL-induced hypercoagulable state and a triggering factor (two-hit theory) [7], and a non-thrombotic mechanism, which is presumably caused by aPL-induced endothelial cell dysfunction [11–16], have been suggested. Complement activation also has a pathogenic role in thrombotic APS, aPL-induced thrombosis, and endothelial cell injury [17, 18]. In our patient, complement levels were marginally decreased, which indicated complement activation as reported by Oku et al. [19] and Barratt-Due et al. [20]. As per the findings from the CAPS Registry by Cervera et al. [1], the most common triggering factor for CAPS is infection (46.7%), which is more prevalent in children than adults according to Berman et al. [21]. In our patient, CAPS may have been triggered by acute pyelonephritis under her untreated APS.

Ocular involvement as an initial presentation of CAPS has been rarely reported [3, 22]. Vaso-occlusive retinopathy is a microangiopathy with diffuse capillary non-perfusion and small arterial or arteriolar occlusions in the retina, which has a very poor visual prognosis due to the high rate of neovascularization and/or vitreous hemorrhage [23]. Pathological findings in vaso-occlusive retinopathy are microthrombosis and immune complex-mediated vasculopathy [24]. Thus, the vaso-occlusive retinopathy in our patient could be considered a usual finding of TMA in APS.

Neuroretinitis is a descriptive term for optic neuropathy that is characterized by optic disc swelling with macular exudates. It is related to abnormal permeability of capillaries deep within the optic disc caused by an infectious process or inflammation. Only one other case of neuroretinitis with a non-infectious origin as an initial manifestation of APS/CAPS has been reported [3]. In the previous case and in ours, there was no evidence of occlusion of the optic disc vasculature and visual function was restored with appropriate treatment. Therefore, in contrast to the vaso-occlusive retinopathy caused by thrombotic pathophysiology, neuroretinitis in this case was presumably caused by a non-thrombotic aPL-induced endothelial cell dysfunction, which has been suggested to be involved in the development of non-thrombotic renal, cerebral, and cardiac lesions in APS patients [25].

In summary, this is a report of a patient with CAPS who initially presented with a rare ocular involvement showing APS-related thrombotic and non-thrombotic microangiopathies. Early diagnosis and timely intervention were crucial for the maintenance of visual function and the survival of this patient.

## Data Availability

All data supporting the conclusions of this article are included in this published article.

## Abbreviations

CAPS: Catastrophic antiphospholipid syndrome
APS: Antiphospholipid syndrome
aPL: Antiphospholipid antibodies
TMA: Thrombotic microangiopathy
LA: Lupus anticoagulant
anti-β2-GPI: Anti-beta2-glycoprotein I
